# Perceived stress is associated with primary dysmenorrhea in Brazilian women: a cross-sectional study

**DOI:** 10.1186/s12889-025-21804-6

**Published:** 2025-04-05

**Authors:** Pâmela Calixto de Moraes, Mariana Arias Avila, Caren Beatriz Firão, Vanessa Patrícia Soares de Sousa, Patricia Driusso

**Affiliations:** 1 Women’s Health Research Laboratory, Physical Therapy Department, Universidade Federal de São Carlos, Rod. Washington Luís km 23, SP-310 São Carlos, SP Brazil; 2 Study Group on Chronic Pain, Laboratory of Research on Electrophysical Agents, Physical Therapy Department, Universidade Federal de São Carlos, São Carlos, SP Brazil; 3 Study and Research Group in Women’s Health Physiotherapy, Faculty of Health Sciences of Trairi, Federal University of Rio Grande do Norte, Santa Cruz, RN Brazil

**Keywords:** Menstrual cycle, Quality of life, Women’s Health

## Abstract

**Objective:**

to evaluate the association between perceived stress and Primary Dysmenorrhea (PD) in Brazilian women.

**Methods:**

We used data from 2,505 participants, a prospective cohort of Brazilian women. The eligibility was restricted to women who had their last three periods and were over 18 years, the exclusion criteria was 1) women with secondary dysmenorrhea and that did not have a period. We measured stress with the Perceived Stress Scale; the interference of PD with the Dysmenorrhea Symptom Interference, and the PD with the Numerical Rating Score. This study used the chi-square test (χ²) to assess associations between perceived stress and binary logistic regression, considering odds ratios (OR) with 95% confidence intervals (CI). All statistical tests were two-tailed, with a significance level of *p* ≤ 0.05.

**Results:**

The average perceived stress score was 25.1 ± 6.6, and the average menstrual pain in the last period was 5.1 ± 2.8. Women with PD are 2.8 (95% CI 1.9 to 4.1) times more likely to have perceived stress and in women with moderate to severe interference of PD, there was 4.8 (95% CI 2.72 to 8.60) increase in the chance to report perceived stress.

**Conclusions:**

Women with PD have higher rates of moderate to high stress. The higher the intensity of menstrual pain, the greater the number of Brazilian women who report mild to high stress.

**Supplementary Information:**

The online version contains supplementary material available at 10.1186/s12889-025-21804-6.

## Introduction

Menstrual health is a crucial component of overall female health, as it can significantly affect their physical, mental, and social well-being. Many women report symptoms associated with menstruation [1, 2], such as dysmenorrhea, back pain, headaches, fatigue, and psychological complaints like mood swings, anxiety, depression, and exhaustion [2].

Primary Dysmenorrhea (PD) is characterized by pelvic pain that radiates to the lower back and thighs, occurring before or during menstruation in the absence of pelvic disease [3]. Women under the age of 25 are twice as likely to develop PD [4]. In Brazil, 62% of women reported experiencing menstrual pain during their last menstrual cycle [5]. One in three women indicated that menstrual symptoms led to a reduction in their daily activities [1, 2].

Menstrual symptoms are common issues that affect most women, causing pain and limiting their activities, which can lead to absenteeism [6, 7]. Women who manage to maintain their activities during menstruation often experience reduced performance, concentration, and ability to study or work compared to other times in their menstrual cycle, directly impacting their academic or professional lives [7].

Although the psychosocial risk factors of PD have not been extensively studied, there appears to be an association between PD, depression, anxiety, and stress [8, 9]. However, studies evaluating this association have used various methods to measure stress, with perceived stress being the most common [10]. Perceived stress refers to an individual’s ability to report their feelings and sensations due to the impact of stress on their daily activities [10].

Perceived stress has been associated with PD in women from various countries, including China [11–13], Saudi Arabia [14], South Korea [15], Japan [16], Iran [17–18], and Jordan [19]. Cultural aspects in countries where menstruation is approached with greater taboo can lead to stigma, shame, or misinformation among women, resulting in higher levels of PD [7]. In Brazil, there is currently no data that establishes a relationship between PD and perceived stress. So, this study aims to evaluate the association between perceived stress and PD in Brazilian women.

## Methods

### Study design

This cross-sectional study was conducted between December 2022 to June 2023 and was carried out in the Women’s Health Research Laboratory (LAMU) at the Universidade Federal de São Carlos and followed the STROBE checklist [20].

This study was disclosed on social media and other networks (Instagram, Facebook, LinkedIn, and WhatsApp) and generated on Google Forms following the approval of the institution’s Research Ethics Committee (Research Ethics Committee of the Federal University of São Carlos, CAAE: 63941722.0.0000.5504), and all methods adhered to the Declaration of Helsinki. All women accepted the Informed Consent Form and selected ‘I agree to participate in this study’ on Google forms to join the research. Subsequently, the participants received a copy of the Informed Consent Form.

All the participants answered a Google form about their sociodemographic characteristics, gynecological, obstetric personal history and about their personal life (if caring for children under 3 years old, if working outside home and if they have someone to help clean the house) that was developed by the authors. The participants also answered valid scales, such as the Numerical Rating Scale (NRS) [21], the Dysmenorrhea Symptom Interference Scale (DSI) [22], and the Perceived Stress Scale (PSS) [23].

Eligibility was restricted to women who had their last three periods and were over 18 years of age. Exclusion criteria included (1) women with secondary dysmenorrhea, (2) Pregnant women, (3) Postpartum women, (4) Breastfeeding women, and (5) post-menopausal women.

### Pain intensity

Pain intensity was measured using the Numerical Rate Scale (NRS) [21]. Participants were asked, “In your last period, how would you rate your menstrual pain?” The answers ranged from zero (absence of pain) to 10 (the worst imaginable pain). This scale has demonstrated validity and reliability in the test-retest situation for assessing menstrual pain related to PD [24]. We classify the NRS score as follows: “No PD” (0 point), “Mild PD” (1 to 3 points), “Moderate PD” (4 to 6 points), “Severe PD” (7 to 10 points) [24].

### Dysmenorrhea symptom interference

Dysmenorrhea Symptom Interference Scale (DSI) measures how dysmenorrhea symptoms interfere with physical, mental, and social activities [22]. It is a reliable, valid, and responsive tool to detect menstrual pain improvement [25]. The DSI has two versions on- and off-menses versions of recall periods.

The on-menses version asked participants to recall their previous 24-hour experiences, and the off-menses version asked participants to identify their experiences from their last menstrual period. Both versions had the same eight items with response options of 1 (not at all) to 5 (very much). Individual item scores were calculated through the averages to generate a total scale score (possible range 1–5). Higher scores indicate greater interference in the quality of life in women with PD (more negative outcome). The DSI total score is “No interference” (1 point), “Mild interference” (1.01 to 2.50 points), “Moderate interference” (2.51 to 3.50 points) and “Severe interference” (3.51 to 5 points) [25].

### Perceived stress

The study measured perceived stress using Cohen’s Perceived Stress Scale (PSS-10), which consists of 10 questions that address control over the demands of daily life [23]. Reis et al. [26] validated the PSS 10-item to Portuguese/Brazil. The PSS questions rate the frequency of their feelings and thoughts related to events and situations in the last month, and each item is rated on a five-point Likert-type scale (1 = never to 5 = very often). Six items are negatively worded (1, 2, 3, 6, 9, 10), while four are positive (4, 5, 7, 8). The positive items are reverse scored before summing all items, resulting in a score ranging from 0 to 40, with higher scores indicating greater stress [26].

The measure scale showed good reliability for both internal consistency and test–retest stability indexes and evidence supporting its construct validity. These findings support the use of the PSS-10 in stress-related research with adults [26]. The interquartile range was used to establish the cut-off point, where a score of ≤ 22 indicated mild symptoms, 23–36 indicated moderate symptoms, and ≥ 37 indicated severe symptoms [19, 26].

### Sample size

According to the Brazilian Institute of Geography and Statistics (IBGE), in 2021, women constituted 51.1% of the total population, 108.7 million individuals. Of this, 75.1% were over 18 years old. The sample size was calculated on the https://pt.surveymonkey.com/mp/sample-size-calculator/ website. The following data were entered: population of Brazilian women aged 18 years or more (81,525,000 women), according to the IBGE, a confidence interval of 95%, and a margin of error of 2%, resulted in a sample of 2,401 women.

### Statistical analyses

Data were analyzed using SPSS software. Statistical frequency analyses of all study variables were conducted. The chi-square (χ²) test assessed associations between perceived stress, pain intensity, pain interference, and menstrual symptoms, based on previous literature [14, 19]. Binary logistic regression modeling tested associations between independent (PPS-10 score ≤ 22 vs. >23) and dependent variables (age group, body mass index, menarche age, onset of PD, married/living with a partner, pain intensity, PD, PD interference, duration of menstruation, length of menstrual cycle, other diagnoses, schooling, work outside, number of pregnancies, number of children living with you, caring for children under 3 years old and have helping at home). Odds ratios (OR) with 95% confidence intervals (CI) estimated the strength of associations. All statistical tests were two-tailed, with a significance level of *p* ≤ 0.05.

## Results

A total of 3,797 participants answered our forms survey between December 2022 and June 2023. Among the respondents, 103 individuals completed the form twice, and their initial responses were excluded. Another 1,189 participants were excluded because they did not meet the eligibility criteria, 668 of these were excluded because they had a medical diagnosis of secondary dysmenorrhea. The final sample for analysis consisted of 2,505 women.

Participants were generally young (mean age 28.8 ± 7.0; 53.3% were under 29 years old). They had a mean body mass index of 25.1 ± 7.4 kg/m² (range 18 to 52). A majority of 53.7% (1,345) were not married or living with a partner, and 83.1% (2,080) had more than 12 years of schooling. Approximately 42.7% of the women (1,068) did not use contraceptive method, and 71.1% (1,755) had never been pregnant before (Table 1).

**Table 1:** Characterization of participants

Variable	Frequency	%
**Age Group**		
18 to 29 years	1460	58.3%
30 to 39 years	804	32.1%
≥ 40 years	241	9.6%
**Body Mass Index (kg/m²)**		
Eutrophic (18.6 to 24.9 kg/m²)	1237	49.3%
Overweight (≥ 25 to 29.9 kg/m²)	678	27.1%
Obesity (≥ 30 kg/m²)	590	23.6%
**Menstruation**		
Participants who completed the questionnaire during their menstrual period.	576	23%
Participants who answered the questionnaire outside of the menstrual period.	1930	77%
**Duration of Menstruation**		
Less than 3 days	192	7.7%
4 to 6 days	1895	75.7%
More than 6 days	417	16.7%
**Length of Menstrual Cycle**		
Irregular Cycle	408	16.3%
Less than 28 days	337	13.5%
About 28 days	1342	53.6%
More than 28 days	417	16.7%
**Onset of Menstrual Cramps in Adolescence**	1882	75.2%
**Married/Living** **With a Partner**	1160	46.3%
**Schooling**		
Up to 4 years	5	0.2%
Up to 9 years	12	0.5%
Up to 12 years	408	16.3%
> 12 years	2080	83.1%
**Contraceptives**		
Do not use	1068	42.7%
Oral hormonal contraception (contraceptive)	517	20.6%
Silver/Copper IUD	219	8.7%
Hormonal IUD	60	2.4%
Injectable	53	2.1%
Condom	571	22.8%
Implant (subdermal contraceptive)	17	0.7%
**Others Diagnostics**		
Diabetes	8	0.4%
Hypertension	35	1.7%
Depression	54	2.7%
Anxiety	487	24.1%
Mood disorders	38	1.9%
**Number of Pregnancies (Including miscarriages)**		
None	1755	70.1%
1	370	14.8%
2	212	8.5%
3	108	4.3%
4 or more	60	2.4%
**Caring For Children Under 3 Years Old**	262	10.5%
**Work Outside**	1744	69.6%
**Have Someone to Help at Home**	1424	56.9%

The average perceived stress score was 28.8 ± 7.0. The interference average was 2.6 ± 0.8. The average for dysmenorrhea in the last period was 5.1 ± 2.8 (Table 2).

**Table 2:** Mean and frequency of the health indicators

Variable	Frequency	%	Mean ± SD
**Perceived Stress (PSS-10)**			28.8 ± 7.0
Mild Stress (≤ 22)	822	32.8%	
Moderate to Severe Stress (≥ 23)	1682	67.2%	
**Pain Intensity (NRS)**			5.1 ± 2.8
No pain (No PD - NRS 0)	217	8.7%	
Mild PD (NRS 1 to 3)	546	21.8%	
Moderate PD (NRS 4 to 6)	786	31.4%	
Severe PD (NRS 7 to 10)	955	38.1%	
**PD Interference (DSI) ** **all participants**			2.6 ± 0.8
No interference (1 point)	74	2.9%	
Mild interference (1.01 to 2.50 points)	1182	47.3%	
Moderate interference (2.51 to 3.50 points)	826	33%	
Severe interference (3.51 to 5 points)	420	16.8%	
**PD Interference (DSI) ** **related on the menstrual period**			1.5 ± 0.8
No interference (1 point)	23	4%	
Mild interference (1.01 to 2.50 points)	294	51%	
Moderate interference (2.51 to 3.50 points)	169	29.3%	
Severe interference (3.51 to 5 points)	90	15.6%	
**PD Interference (DSI) ** **out the menstrual period**			1.6 ± 0.8
No interference (1 point)	50	2.6%	
Mild interference (1.01 to 2.50 points)	891	46.2%	
Moderate interference (2.51 to 3.50 points)	658	34.1%	
Severe interference (3.51 to 5 points)	330	17.1%	

^Abbreviations: PSS−10: Perceived Stress Scale; NRS: Numerical Rate Scale; DSI: Dysmenorrhea Symptom Interference; PD: Primary Dysmenorrhea^

The analysis showed that there was a significant association between the intensities of PD and Perceived Stress. The association analysis showed that there was a significant association between stress and PD interference (Table 3).

**Table 3:** Associations between Pain intensity and perceived stress and DSI

		^PSS−10/^ ^NRS^	^Mild Stress^ (≤ 22)		^Moderate to Severe Stress^ (≥ 23)			^*p*−value^	
		^**No PD** (no pain)^	^116 (53.5%)^		^101 (46.5%)^			^<0.001^	
^**NRS**^		^**Mild PD** (1 to 3)^	^229 (41.9%)^		^317 (58.1%)^				
		^**Moderate PD** (4 to 6)^	^240 (30.5%)^		^546 (69.5%)^				
		^**Severe PD** (7 to 10)^	^237 (24.8%)^		^718 (75.2%)^				
^**DSI**^	^**No Interference** (1 point)^			^65 (62.5%)^		^39 (37.5%)^	^<0.001^		
	^**Mild Interference**^ (1.01 to 2.50 points)			^549 (32.7%)^		^1128 (67.3%)^			
	^**Moderate to Severe Interference**^ (2.51 to 5 points)			^208 (28.7%)^		^515 (71,3%)^			

Abbreviations: DSI: Dysmenorrhea Symptom Interference; NRS: Numerical Rate Scale; PD: Primary Dysmenorrhea

Following the association analysis, logistic regression analysis was used to identify variables associated with perceived stress, categorized as mild (scores ≤ 22) and moderate to severe (scores ≥ 23). Age, BMI, age at menarche, onset of PD in adolescence, pain intensity, presence of PD, PD interference, number of days of menstruation, other diagnoses, and working outside the home were significantly associated with perceived stress. Conversely, the number of pregnancies, the number of children living together, caring for children aged ≤ 3 years, and having help with household chores did not exhibit significant associations with perceived stress (Table 4).

**Table 4:** Univariate and multivariate regression of factors associated with perceived stress among women

Variables		Univariate model			Multivariable Model	
	OR	95% CI	*p*-value	OR	95% CI	*p*-value
**Age Group**						
18–29*						
30–39	0.66	0.55–0.79	**< 0.001**	0.73	0.58–0.91	**0.05**
≥ 40	0.51	0.38–0.68	**< 0.001**	0.63	0.44–0.89	**0.01**
**BMI**						
Eutrophic*.(18.6 to 24.9 kg/m²)						
Overweight (≥ 25 to 29.9 kg/m²)	1.06	0.868–1.312	0.53	-	-	-
Obese (≥ 30 kg/m²)	1.29	1.028–1.636	0.28	-	-	-
**Menarche age group**						
≤ 10*						
11–15	0.69	0.47–0.78	**< 0.001**	0.70	0.51–0.94	**0.02**
≥ 16	0.29	0.12–0.70	**< 0.001**	0.38	0.13–1.06	0.06
**Onset of PD in adolescence**	1.62	1.35–1.96	**< 0.001**	1.11	0.87–1.42	0.38
**Married/Living with a partner**	1.06	0.90–1.26	0.46	-	-	-
**Pain Intensity**						
0 No*						
1–3 Mild	1.59	1.16–2.18	**< 0.001**	1.43	0.99–2.06	**0.05**
4–6 Moderate	2.61	1.92–3.55	**< 0.001**	2.09	1.46–3.02	**< 0.001**
7–10 Severe	3.48	2.56–4.72	**< 0.001**	2.50	1.74–3.61	**< 0.001**
**PD** NRS ≤ 3 (no PD)*						
NRS ≥ 4 (PD)	2.18	1.83–2.61	**< 0.001**	2.78	1.90–4.10	**< 0.001**
**PD Interference**						
No *						
Mild	3.42	2.27–5.15	**< 0.001**	3.04	1.84–5.03	**< 0.001**
Moderate to Severe	4.61	2.97–7.13	**< 0.001**	4.83	2.72–8.60	**< 0.001**
**PD Interference**	2.60	1.52–4.42	**< 0.001**	3.35	1.73–6.51	**< 0.001**
**Duration of Menstruation (in days)**						
Less than 3*						
4–6	1.13	0.83–1.54	0.42	1.07	0.75–1.52	0.70
More than 6	1.74	1.21–2.51	**< 0.001**	1.46	0.95–2.22	0.08
**Length of Menstrual Cycle**						
Irregular cycle*						
Less than 28 days	0.61	0.44–0.84	**< 0.001**	0.73	0.50–1.05	0.09
About 28 days	0.63	0.49–0.81	**< 0.001**	0.80	0.60–1.07	0.14
More than 28 days	0.63	0.46–0.85	**< 0.001**	0.72	0.51–1.02	0.06
**Other diagnoses**						
None*						
Diabetes	0.72	0.18–2.88	0.64	0.64	0.15–2.72	0.54
Hypertension	1.08	0.54–2.13	0.83	1.34	0.65–2.76	0.42
Anxiety	2.10	1.67–2.64	**< 0.001**	1.90	1.50–2.41	**< 0.001**
Depression	2.05	1.10–3.81	**0.02**	2.14	1.11–4.04	**0.02**
Mood Disorder	3.18	1.39–7.28	**< 0.001**	2.63	1.13–6.12	**0.02**
**Schooling**						
Up to 4 years*						
Up to 9 years	3.66	0.17–77.55	0.40	0.58	0.20–16.48	0.75
Up to 12 years	1.14	0.12–11–14	0.90	1.02	0.10–10.27	0.98
> 12 years	0.61	0.64–5.96	0.67	0.69	0.07–6.83	0.75
**Work outside**	0.72	0.60–0.87	**< 0.001**	0.84	0.68–1.05	0.12
Number of Pregnancies*						
None						
1	1.26	0.99–1.62	0.59	-	-	-
2	0.88	0.65–1.18	0.40	-	-	-
3	1.03	0.68–1.56	0.87	-	-	-
4	1.17	0.66–2.05	0.58	-	-	-
**Number of children ** living with you*						
None *						
1	1.21	0.95–1.55	0.12	-	-	-
2	0.84	0.623–1.14	0.28	-	-	-
3	1.66	0.886–3.10	0.11	-	-	-
4 more	0.54	0.52–3.43	0.54	-	-	-
**Caring For Children Under 3 Years Old**	1.19	0.904–1.58	0.21	-	-	-
**Have Helping at Home**	1.06	0.900–1.26	0.46	-	-	-

Abbreviations: BMI: Body Mass Index; PD: Primary Dysmenorrhea; * reference category

Subsequent to the univariate regression analysis, variables demonstrating significant associations were incorporated into the multivariate regression analysis. During the multivariate regression analysis, these variables no longer exhibited significant associations: onset of PD in adolescence, duration of menstruation, length of menstrual cycle, specific conditions from other diagnoses, and engagement in physical exercise (Table 4).

Multivariate logistic regression analysis revealed a significant association between perceived stress and several variables, but not others. Age, age at menarche, body mass index (BMI), presence of PD, severity of PD, and interference of PD, indicating a heightened likelihood of perceived stress. However, the number of days in menstruation, number of days in the menstrual cycle, and working outside the home were not significantly associated with perceived stress and were therefore excluded from the multivariate model.

## Discussion

In this cross-sectional study, conducted with Brazilian women, we aimed to evaluate the association between perceived stress and PD. We also analyzed how the severity of PD and interference in physical, social, and mental activities have affected the stress level of women with or without PD.

Our study found that 69,5% of participants reported experiencing primary dysmenorrhea, the prevalence may vary according to the country, and the evaluation method for classifying PD, and their mean pain score is 5.1 ± 2.8 using the Numeric Rating Scale (NRS) and a mean Perceived Stress Scale-10 (PSS-10) score of 28.8 ± 7.0. The perceived stress and intensity of menstrual pain in Brazilian women, showed a significant association, with an increase in the percentage of moderate to severe stress as the severity of PD increased. Our data were consistent with findings in literature, showing that women who claimed PD had higher levels of perceived stress compared to women who not claimed PD [4, 8, 13].

Our findings show that women with moderate to severe PD are 2 to 2.5 times more likely to have perceived stress. This aligns with existing research demonstrating that women experiencing chronic stress tend to report higher levels of pain and greater menstrual pain intensity, particularly within the moderate-to-severe range [4, 9–27].

Studies focusing specifically on PD and stress in women provide crucial support for our findings and underscore the intricate connection between psychological well-being and the severity of menstrual pain.

The association between stress and PD is biologically plausible because, during stressful situations, our body undergoes a cascade of neuroendocrine responses [13]. The hypothalamus initiates this process by mediating the secretion of adrenocorticotropic hormone (ACTH), which subsequently increases cortisol levels. Elevated cortisol levels may inhibit the release of follicle-stimulating hormone (FSH) and luteinizing hormone (LH), potentially impairing follicle development. This can lead to altered progesterone synthesis and release, which in turn sensitizes the synthesis of prostaglandins PGF2α and PGE2 and their receptors in the myometrium, ultimately contributing to PD [13, 28].

When evaluating the impact of the menstrual pain on social and mental activities, we found that 33% of participants experienced moderate interference in their activities due to PD, while 47.3% reported only slight interference. Our analysis revealed a significant association between perceived stress and the extent of activity interference caused by PD in Brazilian women. Participants who reported greater interference in their activities also exhibited higher levels of moderate to high perceived stress.

In the multivariate regression analysis, we observed that interference from PD was significantly associated with perceived stress. Women experiencing mild to moderate and severe interference from PD symptoms had nearly 3 to 4 times higher odds of reporting perceived stress. Our findings are consistent with existing research, which indicates that women with PD may experience reduced productivity and physical well-being during their menstrual period. This can lead to increased absenteeism from work and academic activities. Additionally, PD has been shown to negatively impact young women’s academic performance, particularly in tasks requiring high levels of concentration during the menstrual phase [4, 7, 10].

Other findings of this study pertained to the age of the women, with 53% of the participants being between 18 and 29 years old. According to the literature, women up to 25 years of age are twice as likely to have PD [4]. Furthermore, biopsychosocial factors, which vary across different age groups, may influence not only the prevalence but also the perception of pain. Younger women often report greater pain intensity, due to heightened hormonal fluctuations, increased pain sensitivity, and emotional factors [5, 17, 19].

Age and menarche age demonstrated a significant association with perceived stress. Women aged between 30 and 39 years old demonstrated a 27% lower chance of perceived stress than compared to women aged 18–29 years. The age of menarche was associated with a 30% reduction in the chance of having perceived stress in girls who menstruated for the first time between 11 and 15 years old; the data suggests that with an increase in the age of menarche, there is a corresponding decrease in stress levels. Some authors hypothesize that the earlier menarche occurs, the greater the possibility of PD, due to the longer period in which the body is exposed to menstruation and, consequently, to prostaglandin [29].

This study’s strengths lie in its use of well-defined construct scales validated for the Brazilian population, the multivariate regression model used for data analysis helps identify interactions and relationships between different variables related to pain and the severity of PD, providing a more comprehensive view of the data. Additionally, it is pioneering in its focus, as, to our knowledge, no previous studies have evaluated the relationship between stress and PD in Brazilian women.

However, this study has limitations and potential confounding factor. The use of an online questionnaire includes only women with internet access, which may have influenced the high number of participants with a high level of education in this study. Pain and stress are multifactorial phenomena, and our study was unable to account for other variables that might influence participants’ perceptions of these factors. Furthermore, some participants completed the questionnaire during their menstrual period, while others did so outside of it. This variation could have influenced the assessment of dysmenorrhea, as the timing in relation to the menstrual cycle may affect pain perception.

In conclusion, our study finds a significant association between PD and perceived stress in Brazilian women. Women with PD were twice as likely (a two-fold increase) to report perceived stress compared to those without PD. Furthermore, we found that the severity of PD was positively correlated with the percentage of moderate to high stress reported by Brazilian women. It is important to highlight that the present study is a cross-sectional study, and the analysis of association can identify relationships between variables, but it does not establish cause and effect.

### Implications for practice and/or policy

To our knowledge, this study is the first to examine the association between perceived stress and Primary Dysmenorrhea (PD). Our findings highlight the need for a paradigm shift in research focus, prompting future studies to explore not only pain-relieving interventions but also the moderating effects of stress on PD severity. By broadening the understanding of menstrual pain in Brazilian women to encompass stress as a potential contributing factor, we can enhance the effectiveness of multidisciplinary teams in addressing both the physical and emotional symptoms associated with PD.

Additionally, these findings have significant implications for the development of public health policies and the enhancement of interactions between healthcare professionals and patients. Recognizing stress as a factor influencing the severity of PD highlights the importance of integrated care approaches that prioritize open communication, patient education, and personalized treatment plans. This approach can result in more effective treatments, enhance the quality of life for women with PD, and bolster broader public health initiatives centered on women’s health.

## Electronic supplementary material

Below is the link to the electronic supplementary material.

Supplementary Material 1

## Data Availability

The data can be accessed by requesting from the corresponding author.

## Abbreviations

PD: Primary Dysmenorrhea
PSS-10: Perceived Stress Scale
NRS: Numerical Rate Scale
DSI: Dysmenorrhea Symptom Interference
IBGE: Brazilian Institute of Geography and Statistics
CI: Confidence Intervals
OR: Odds ratios
BMI: Body Mass Index
IUD: Intrauterine Device
