# Supplementary material for: Perceived stress is associated with primary dysmenorrhea in Brazilian women: a cross-sectional study
Source: BMC Public Health. 2025 Apr 5;25:1290. doi: 10.1186/s12889-025-21804-6 (PMC11971888; doi:10.1186/s12889-025-21804-6)
Supplement: Supplementary file 1 — Supplementary Material 1 [file 12889_2025_21804_MOESM1_ESM.pdf]

Assessment Form for Sociodemographic and Gynecological Health

Are you under 18 years old, or pregnant, or up to 6 months postpartum, or breastfeeding, or in menopause, or do you have a medical diagnosis of endometriosis?
( ) Yes
( ) No

In which Brazilian state do you currently reside?
(Mark only one)
( ) Acre
( ) Alagoas
( ) Amapá
( ) Amazonas
( ) Bahia
( ) Ceará
( ) Brasilia
( ) Espírito Santo
( ) Goiás
( ) Maranhão
( ) Mato Grosso
( ) Mato Grosso do Sul
( ) Minas Gerais
( ) Pará
( ) Paraíba
( ) Paraná
( ) Pernambuco
( ) Piauí
( ) Rio de Janeiro
( ) Rio Grande do Norte
( ) Rio Grande do Sul
( ) Rondônia
( ) Roraima
( ) Santa Catarina
( ) São Paulo
( ) Sergipe
( ) Tocantins
( ) I don’t know/I don’t want to answer"

What is your age? ____________

Marital status:
( ) In a conjugal relationship (married or in a stable union)
( ) Not in a conjugal relationship (single, widowed, or divorced)
( ) I don't know/I don't want to answer"

What is your education level?
( ) Up to 4 years (Elementary school)
( ) Up to 9 years (High school)
( ) More than 9 years (Higher education)

From the items below, do you have any gynecological medical diagnosis?
(Mark all that apply)

( ) I have no diagnosis
( ) Endometriosis
( ) Polycystic ovary
( ) Fibroid
( ) Uterine/ovarian/vulvar cancer
( ) Vulvar edema
( ) Urogynecological infections (e.g., sexually transmitted diseases)
( ) Genital malformations
( ) Uterine prolapse (descent of the uterus)
( ) Vulvodynia (burning and/or pain in the genital area)
( ) Adenomyosis (thickening within the uterine walls)

From the items below, do you have any medical diagnosis?
(Mark all that apply)
( ) I have no diagnosis
( ) Hypertension
( ) Anxiety
( ) Depression
( ) Mood disorder

Do you continuously use any medication?
(Mark only one)
( ) Yes
( ) No
( ) I don’t know/I don’t want to answer

Which of these medications do you use?
(Mark all that apply)
( ) Anxiolytic
( ) Antidepressant
( ) Contraceptive
( ) Antihypertensive
( ) I don’t know/I don’t want to answer

Have you menstruated in the last 3 months? (You can still answer the questionnaire even if the answer is no).
( ) Yes
( ) No

At what age did you have your first menstruation?
(Mark only one)
( ) 8 years
( ) 9 years
( ) 10 years
( ) 11 years
( ) 12 years
( ) 13 years
( ) 14 years
( ) 15 years
( ) 16 years
( ) 17 years
( ) 18 years
( ) 19 years
( ) I don’t know/I don’t want to answer

What is the average duration of your menstrual cycle? (By cycle, we mean the period between one menstruation and the next, that is, from the first day of menstruation to the first day of the next menstruation.)
(Mark only one)

( ) Less than 28 days
( ) About 28 days
( ) More than 28 days
( ) My cycles are irregular

How many days does your menstruation last?

(Mark only one)

( ) Less than 3 days
( ) Between 4 and 6 days
( ) More than 6 days

Are you currently using any form of contraception?

(Mark all that apply)

( ) Copper IUD or copper with silver
( ) Hormonal IUD (e.g., Mirena or Kyleena)
( ) Hormonal injectable contraception
( ) Oral hormonal contraception (birth control pill)
( ) Female/male condom
( ) Implants
( ) I am not using any

Was the contraceptive method used prescribed by a doctor?
(Mark only one)
( ) Yes
( ) No
( ) I don't know/I don't want to answer

Do you have menstrual cramps?
( ) Yes
( ) No

Did your cramps start during adolescence?
( ) Yes
( ) No

What symptoms do you associate with your premenstrual (days before menstruation) / menstrual period, considering your last menstruation?

	Yes	No
Abdominal cramps		
Headache/Migraine		
Diarrhea		

Nausea		
Discomfort		
Irritability		
Change in appetite		
Abdominal bloating		
Breast swelling or pain		
Decreased sleep quality		
Acne (pimples) or worsening of dermatological issues		
Dizziness		
Ringing in the ears		
More emotional		
Difficulty concentrating		
Increased anxiety		
Low self-esteem		
Leg pain		
Lower back pain		
Joint pain		

Do you use any medication to alleviate menstrual cramps?
(Mark only one)

( ) Yes
( ) No
( ) I don't know/I don't want to answer

After using the medication, the pain:
(Mark only one)
( ) Does not change
( ) Shows slight improvement
( ) Shows significant improvement
( ) Is completely resolved
( ) I don't know/I don't want to answer

How many pregnancies have you had (including miscarriages)?
(Mark only one)
( ) None
( ) 1
( ) 2
( ) 3
( ) 4
( ) 5 or more
( ) I don’t know/I don’t want to answer

Have you ever had a miscarriage?
(Mark only one)
( ) Yes
( ) No
( ) I don’t know/I don’t want to answer

Are you currently breastfeeding?
( ) Yes
( ) No
( ) I don’t know/I don’t want to answer

Have you ever had a multiple pregnancy? (Pregnancy with more than one baby – twins, triplets)
(Mark only one)
( ) Yes
( ) No
( ) I don’t know/I don’t want to answer

What types of delivery have you had?
(Mark all that apply)
( ) None
( ) Vaginal/Normal
( ) Cesarean
( ) Vaginal/Normal and Cesarean
( ) I don’t know/I don’t want to answer

How many children currently live with you?
( ) 1
( ) 2
( ) 3

( ) 4 or more
( ) I don’t know/I don’t want to answer

Do you take care of any child under 3 years old?
( ) Yes
( ) No
( ) I don’t know/I don’t want to answer

Do you work outside the home?
( ) Yes
( ) No
( ) I don’t know/I don’t want to answer

Do you have someone who helps you clean/take care of the house?
( ) Yes
( ) No
( ) I don’t know/I don’t want to answer
